# Self-reported pain severity and use of cannabis and opioids in persons with HIV in an urban primary care setting in Northern California: A cross-sectional study

**DOI:** 10.1097/MD.0000000000037581

**Published:** 2024-03-29

**Authors:** Hannah J. Kim, Derek D. Satre, Wendy Leyden, Amy S. Leibowitz, Cynthia I. Campbell, Michael J. Silverberg

**Affiliations:** a Kaiser Permanente National Patient Care Services, Oakland, CA; b Community Health Systems, University of California, San Francisco, San Francisco, CA; c Kaiser Permanente Northern California Division of Research, Oakland, CA; d Weill Institute for Neurosciences, Department of Psychiatry, University of California, San Francisco, San Francisco, CA; e Department of Psychiatry and Behavioral Sciences, University of California, San Francisco, San Francisco, CA.

**Keywords:** alcohol, cannabis, cross-sectional, opioids, pain, pain management

## Abstract

Persons with HIV (PWH) experience high levels of pain. We examined the relationship of pain severity with use of cannabis and prescription opioids among PWH. This cross-sectional study evaluated associations between self-reported pain (moderate/severe vs mild/none) and cannabis and prescription opioid use in a primary care sample of PWH enrolled in an alcohol use treatment study at Kaiser Permanente, San Francisco. Prevalence ratios (PR) for moderate/severe pain associated with cannabis, opioid use, or both in the prior 30 days were obtained from Poisson regression models. Adjusted models included race/ethnicity, education, employment, HIV ribonucleic acid levels, depression, and anxiety. Overall, 614 PWH completed baseline questionnaires from May 2013 to May 2015, among whom 182/614 (29.6%) reported moderate/severe pain. The prevalence of moderate/severe pain varied by substances: 19.1% moderate/severe pain among study participants who reported neither cannabis or opioids, 30.2% for cannabis alone, 41.2% for opioids alone, and 60.9% for those reporting both substances. In adjusted models, compared with PWH who reported neither substance (reference), prevalence of moderate/severe pain was higher for those using cannabis alone (PR 1.54; 95% CI 1.13–2.09), opioids alone (PR 1.96; 95% CI 1.31–2.94), and those reporting both (PR 2.66; 95% CI 1.91–3.70). PWH who reported opioid and/or cannabis use were more likely to report moderate/severe pain compared with PWH who did not report use of these substances. To improve patient care, it is vital to assess patients’ approaches to pain management including substance use and target appropriate interventions to reduce pain in PWH.

## 1. Introduction

Along with improved long-term survival using anti-retroviral therapy (ART), pain is a common problem for persons with HIV (PWH).^[1]^ The causes of acute and chronic pain in PWH are varied and include infections, injuries or surgery not related to HIV, and side effects of HIV treatment including peripheral neuropathy.^[2]^ Treatments for pain in HIV have included use of prescription opioids^[3]^ and medicinal cannabis.^[4,5]^ Brunet et al reported that 11% of PWH used prescription opioids episodically, while 16% used prescription opioids long-term.^[6]^ Harris and colleagues (2014) found that many PWH used cannabis for recreational purposes, while about 21% used it for medicinal purposes (most commonly to relieve pain and stress [80%]).^[5]^ In the context of increasing cannabis legalization in combination with concern about opioid overdose risks, it is valuable to understand the extent to which PWH experiencing pain use these 2 substances separately and in combination.

This study is one of the first to our knowledge to examine associations between use of both opioids and cannabis by pain severity among PWH. The purpose of this study was to describe the prevalence of moderate/severe pain among those reporting cannabis, prescription opioid use, or both, among PWH enrolled in an alcohol treatment trial, independent of depression, demographics, and other characteristics. We hypothesized that moderate/severe pain would be associated with higher use of both cannabis and prescription opioids.

## 2. Methods

### 2.1. Study setting, design, and participants

This cross-sectional analysis was conducted in Kaiser Permanente Northern California (KPNC), a health system of 4 million members where over 9500 PWH receive care.^[7]^ Study participants included 614 PWH from KPNC San Francisco Medical Center recruited for a randomized clinical trial evaluating behavioral interventions to reduce unhealthy alcohol use, the “Health and Motivation Study” (U01AA021997). Participant enrollment details regarding inclusion criteria and eligibility have been described previously.^[8,9]^ Patients enrolled were part of a randomized clinical trial to reduce unhealthy alcohol use, with eligibility being: having 1 binge drinking episode in the prior 12 months: any days of drinking ≥3 drinks in a day (for women) and ≥4 drinks in a day (for men). Using mailed recruitment letters, flyers, newsletters, and referrals, the study team contacted 2873 PWH between April 25, 2013 through May 29, 2015. A flow diagram of participant screening and enrollment for the main clinical trial can be found in another manuscript.^[8,9]^ The study was also conducted at baseline, prior to any interventions provided. The study team enrolled 614 (79%) of the 773 eligible patients. There were 85 (11%) patients who declined to participate, and 74 (9%) patients did not show for their appointment and were unresponsive thereafter. Randomization and careful allocation concealment was conducted to minimize the potential for selection bias. The KPNC and University of California, San Francisco institutional review boards approved all study procedures.

### 2.2. Measures

The main dependent variable was pain reported at study intake. Participants were asked “How much bodily pain have you had in the past 4 weeks? (none, very mild, mild, moderate, severe, very severe).” Pain was dichotomized into 2 groups: none/mild pain (scores 1–3) and moderate/severe pain (scores 4–6). Sensitivity analyses were completed for pain groups none (score 1) and mild/moderate/severe pain (scores 2–6) (data not reported) to assess if patients with mild pain may behave similarly with regards to the associations with substance use.

#### 2.2.1. Substance use.

Substance use included self-reported cannabis use, prescription opioid use (i.e., Vicodin, Oxycontin, codeine, and morphine), both cannabis and prescription opioid use, or neither substance in the last 30 days. Other substance use (including prescription drug misuse, stimulants, cocaine, painkillers, heroin, hallucinogens, and ecstasy) and tobacco use were also defined as >1 time use in the last 30 days. Weekly unhealthy alcohol use was defined as having 4 drinks for women and 5 drinks for men in a day, on 4 or more days in the last 30 days. Single-item screening questions have been validated for identifying substance use problems in health care settings.^[10]^

#### 2.2.2. Other predictors.

Age, gender (male or female), race/ethnicity (White, Hispanic, Black, unknown/other), marital status (yes or no), education (<High School/High School/General Educational Diploma, Associate of Arts/Associate of Sciences, College, ≥Graduate School), and employment status (yes or no) were obtained from the baseline survey. HIV exposure risk factor (i.e., men who have sex with men (MSM) or bisexual, injection drug use, heterosexual, and unknown/other), CD4 cell counts (continuous), and HIV ribonucleic acid (RNA) levels (i.e., <75 and ≥75 copies/mL) were obtained from the KPNC HIV registry.^[7]^ Current use of ART medications (yes/no), and adherence to medications in last month (≥90% vs <90% of prescribed medications taken) were ascertained by self-report at baseline.

Depression and anxiety were measured using the Patient Health Questionnaire (PHQ-9) and the generalized anxiety disorder (GAD-7), respectively. The PHQ-9, based on DSM-IV depression criteria, is a valid and reliable measure of depression severity.^[11]^ PHQ-9 scores range from 0 to 27, with higher scores indicating more symptoms; scores 10 to 27 indicate moderate/severe depression.^[12]^ Anxiety was measured using GAD-7, a valid and reliable measure of anxiety.^[13]^ GAD-7 scores range from 0 to 21; scores 10 to 21 indicate moderate/severe anxiety.

### 2.3. Analyses

Participant characteristics were examined descriptively in the total sample (Table 1). Unadjusted and adjusted prevalence ratios (PR) for the outcome of moderate/severe pain (vs mild/none) were obtained from Poisson regression models with robust standard errors using Proc Genmod SAS Version 9.3 (SAS Inc., Cary, NC). The main predictors were prescription opioid use and cannabis use, categorized as cannabis only, opioids only, both, neither (reference). Adjusted models include variables that were found to be significantly (*P* < .05) associated with moderate/severe pain in unadjusted models (Table 2).

**Table 1:** Baseline characteristics of study sample: persons with HIV, total and by pain.

Characteristic	Total	None/mild pain	Moderate to severe pain
	N = 614	n = 432	n = 182
Substance use			
MJ use	39.4	39.1	40.1
Opioid use	8.3	6.9	11.5
Both	10.4	5.8	21.4
Neither	41.9	48.2	26.9
Age mean (SD) (in model-per 10 years)	48.9 (11.0)	48.3 (11.1)	51.8 (10.4)
Male, %	96.7	97.22	95.6
Race/Ethnicity, %			
White	62.7	63.19	61.54
Hispanic	14	12.5	17.58
Black	9.1	7.64	12.64
Unknown/Other*	14.2	16.67	8.24
Married, %	43.8	43.52	44.51
Education, %			
<HS/HS/GED	25.4	20.8	36.3
AA/AS	17.1	17.1	17
College	35.2	38.2	28
≥Graduate school	22.3	23.8	18.7
Employed, %	72.5	77.8	59.9
HIV exposure risk factor, %			
MSM or bisexual	73.1	72.7	74.2
Injection drug use	6.4	6	7.1
Heterosexual	4.2	4.4	3.9
Unknown/Other†	16.3	16.9	14.8
CD4 cell count mean (SD)	71.8	676.3 (271.3)	666.4 (298.2)
HIV RNA levels <75 copies/mL mean	93.5	94.7	90.7
>90% self-report adherence to ART past 30 days, %	83.9	85.7	79.7
Tobacco use, %‡	24.4	25.5	22
Other substance use‡,§, %	27.4	28.2	25.3
Moderate/severe depressionǁ, %	16.1	11.8	26.4
Moderate/severe anxiety¶, %	13.8	11.1	20.3

Baseline characteristic frequencies are shown for all persons with HIV total (first column), for those who report none/mild pain (second column, scores 1–3), and for those who reported moderate/severe pain (third column, scores 4–6).

AA/AS = associates in arts/associates in science, ART = anti-retroviral therapy, HS/GED = high school/general education development, MSM = men who have sex with men, RNA = ribonucleic acid.

* Includes Asian, American Indian, other, and unknown.

† Includes transfusion/other/unknown.

‡ Use in the past 30 days.

§ Other substances include prescription drug use other than as prescribed, tranquilizers, stimulants, cocaine, painkillers, heroin, hallucinogens, and ecstasy.

ǁ Patient health questionnaire (PHQ-9) score.

¶ Generalized anxiety disorder (GAD-7) score.

**Table 2:** Factors associated with prevalence of moderate to severe pain among persons with HIV.

Characteristic	Unadjusted*			Adjusted		
	PR	95% CI	*P*-value	PR	95% CI	*P*-value
Cannabis/opioid use						
Cannabis use	1.58	(1.15, 2.17)	0.005	1.54	(1.13, 2.09)	0.007
Opioid use	2.16	(1.43, 3.27)	0.000	1.96	(1.31, 2.94)	0.001
Both	3.20	(2.32, 4.40)	<0.0001	2.66	(1.91, 3.70)	<0.001
Neither	1 (ref)			1 (ref)		
Age mean (SD, in model-per 10 yr)	1.23	(1.10, 1.38)	0.000	1.23	(1.09, 1.39)	0.001
Male, %	0.73	(0.42, 1.27)	0.268			
Race/ethnicity, %						
White	1 (ref)			1 (ref)		
Hispanic	1.28	(0.93, 1.75)	0.127	1.37	(1.00, 1.87)	0.050
Black	1.41	(0.99, 2.00)	0.05	1.14	(0.78, 1.66)	0.49
Unknown/other†	0.59	(0.36, 0.96)	0.035	0.59	(0.35, 0.99)	0.047
Married, %	1.03	(0.81, 1.31)	0.822			
Education, %						
<HS/HS/GED	1 (ref)			1 (ref)		
AA/AS	0.70	(0.49, 0.99)	0.04	0.86	(0.61, 1.21)	0.39
College	0.56	(0.41, 0.75)	0.000	0.72	(0.53, 0.97)	0.029
≥Graduate school	0.59	(0.42, 0.83)	0.002	0.67	(0.48, 0.92)	0.014
Employed, %	0.57	(0.45, 0.72)	<0.0001	0.77	(0.60, 0.97)	0.030
HIV exposure risk factor, %						
MSM or bisexual	1 (ref)					
Injection drug use	1.11	(0.70, 1.77)	0.66			
Heterosexual	0.90	(0.47, 1.71)	0.74			
Unknown/other‡	0.90	(0.63, 1.28)	0.55			
CD4 cell count mean (SD)	0.99	(0.95, 1.04)	0.688			
HIV RNA levels <75 copies/mL mean	0.67	(0.46, 0.99)	0.042	0.68	(0.44, 1.05)	0.079
>90% self-report adherence to ART past 30 d, %	0.75	(0.56, 1.01)	0.056			
Tobacco use, %	0.87	(0.65, 1.17)	0.366			
Unhealthy alcohol use, %§	1.11	(0.87, 1.42)	0.403			
Other substance use, %ǁ	0.90	(0.68, 1.19)	0.4564			
Moderate/Severe depression, %¶	1.86	(1.45, 2.39)	<0.0001	1.40	(1.03, 1.92)	0.034
Moderate/Severe Anxiety, %#	1.59	(1.20, 2.10)	0.00	1.17	(0.83, 1.64)	0.38

Unadjusted and adjusted prevalence ratios (PR) with 95% confidence intervals and *P*-values for association of outcome of moderate to severe pain among persons with HIV with primary exposure of substance use (i.e., both prescription opioid use and cannabis use, prescription cannabis use only, prescription opioids use only, neither), as well as other demographic and clinical factors. PRs were obtained from Poisson regression models with robust standard errors using Proc Genmod SAS Version 9.3 (SAS Inc., Cary, NC). In the adjusted model, characteristics which were statistically significant (*P* < .05) in the unadjusted model were included.

AA/AS = associates in arts/associates in science, ART = anti-retroviral therapy, HS/GED = high school/general education development, MSM = men who have sex with men, RNA = ribonucleic acid.

* Based on Pearson chi-square test comparing <50 years old with ≥50 years old.

† Includes Asian, American Indian, other, and unknown.

‡ Includes transfusion/other/unknown.

§ Defined as 4 drinks for women and 5 drinks for men in a day for 4 + days in the past 30 days.

ǁ Use in the past 30 days; other substances include prescription drug use other than as prescribed, tranquilizers, stimulants, cocaine, painkillers, heroin, hallucinogens, and ecstasy.

¶ Patient health questionnaire (PHQ-9) score.

#Generalized anxiety disorder (GAD-7) score.

## 3. Results

Of the 614 PWH in the study sample (Table 1), 96.7% were male, 62.7% were White, with mean age 48.9 years (SD = 11.0) and 72.5% were employed. The population was well-treated with 93.5% HIV RNA levels <75 copies/mL and 71.8% CD4+ T cell counts (≥ 500 cells/mm^3^). Finally, 16.1% had moderate/severe depression, and 13.8% moderate/severe anxiety. At baseline, 40.1% of study participants reported cannabis use, 11.5% opioids, 21.4% both and 26.9% neither substance.

When we conducted sensitivity analysis for pain groups none (score 1) and mild/moderate/severe pain (scores 2–6), we found that results for substance use were attenuated compared with the groups when mild pain was grouped with moderate and severe pain. Given this finding, we concluded that the “mild” group was most similar to the “none” group which resulted in our combining none with the mild pain group.”

**Figure 1.**
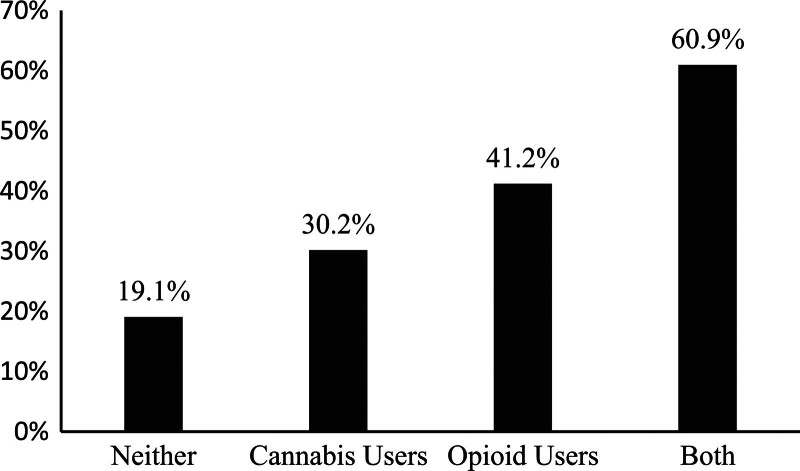
Prevalence of moderate or severe pain by use of cannabis and/or opioids. Bar graphs depicting the percentage of persons with HIV who report moderate or severe pain by cannabis and/or opioid use.

Of the 614 PWH, 182 (29.6%) reported moderate/severe pain and 432 (70.4%) reported none/mild pain. However, in bivariate analyses, the prevalence of moderate/severe pain varied by substances reported at baseline. As shown in Figure 1, we found 19.1% moderate/severe pain among study participants who reported neither cannabis or opioids, 30.2% for cannabis alone, 41.2% for opioids alone and 60.9% for those reporting both substances.

In the final adjusted model (Table 2), PWH who reported moderate to severe pain were 1.54 times (95% CI: 1.13–2.09) more likely to use cannabis alone compared with those who used neither cannabis nor prescription opioids. PWH who reported moderate to severe pain were 1.96 times (95% CI: 1.31–2.94) more likely to use prescription opioids alone compared with those who used neither cannabis nor prescription opioids. PWH who reported moderate to severe pain were 2.66 times (95% CI: 1.91–3.70) more likely to use both cannabis and prescription opioids compared with those who used neither substance.

Several other factors were significant. Older age (PR = 1.23, 95% CI: 1.09–1.39) and greater depression (PR = 1.40, 95% CI: 1.03–1.92) were associated with moderate to severe pain. Factors associated with a reduced prevalence of moderate/severe pain included unknown/other race/ethnicity (PR = 0.59, 95% CI: 0.35–0.99) compared with whites, college degree (PR = 0.72, 95% CI: 0.53–0.97) and graduate school and higher (PR = 0.67, 95% CI: 0.48–0.92) compared with less than high school, and being employed (PR = 0.77, 95% CI: 0.60–0.97). HIV RNA levels < 75 copies/ml was not statistically significant in the model (PR = 0.68, 95% CI: 0.44–1.05). Factors not associated with moderate/severe pain included gender, marital status, HIV exposure risk factor, CD4 cell count, self-report adherence to ART in the past 30 days, tobacco use, alcohol use, and other substance use (Table 2).

## 4. Discussion and implications for practice

Given the high burden of pain in PWH and the need for effective pain management, it is important to understand how PWH use substances in the context of pain.^[14]^ There is some evidence that cannabis may be effective for alleviating pain, and the use of cannabis is likely to continue to increase as a result of legalization of recreational cannabis in California and other states.^[15]^ In our primary care-based sample, we found that PWH who reported moderate/high pain were more likely to report opioid and cannabis use compared to PWH with none/mild pain. These findings highlight the importance of understanding how these substances are used by PWH, to improve pain management and minimize risks to patient health.

Recent studies have found a decrease in pain medication use (both opioids and non-opioids) for Medicare Part D and Medicaid populations in states where medical cannabis laws were approved.^[16]^ Our study observed prescription opioid use in 1 region of California and found prevalent use of cannabis in PWH, approximately 40% in the none to mild pain group and the moderate to severe pain group. This study was conducted when medical cannabis laws were approved in the state of California, yet concerns were growing about opioid use risks, and prescribing policies were becoming more conservative.^[17]^ The legalization of recreational cannabis laws can affect the use of cannabis for pain^[17]^ and future studies should assess how PWH utilize substances other than prescription opioids to manage pain. One research team found that depression, alcohol consumption, and marijuana use were not significantly associated with current pain nor with severe pain.^[18]^ Our study found that depression and marijuana use were significantly associated with pain in the adjusted model. Our definition of unhealthy alcohol use was 4 or more drinks for women and 5 or more drinks for men in a day for 4+ days in the past 30 days, whereas this prior study defined alcohol consumption as either hazardous or nonhazardous as 14 standard drinks per week for men and 9 per week for women.^[18]^ Pain is one of the most common medical reasons for marijuana use,^[14,19]^ and although our study found a significant relationship, future studies are needed to better understand the use of marijuana for pain in PWH. In addition to understanding how PWH use substances to manage pain, it is important for future studies to address depression and to differentiate between chronic pain and neuropathic pain.

Several sociodemographic factors were associated with pain in our sample, including age, unknown/other race/ethnicity, education, and employment. These results are also of interest in understanding those most in need of assistance in managing pain. Lawson et al^[2]^ (2015) found that among PWH in the UK, increasing age was an independent risk factor of pain. Our study also found that older age was a risk for moderate to severe pain. Older PWH report poorer physical quality of life^[20]^ and thus our findings also suggest that pain should be an important consideration in the aging population. Joseph et al^[18]^ found that the odds of having any pain was 2.49 times greater in PWH who reported anxiety. In addition to anxiety, holding multiple stressors and marginalized identities, such as being African American, impact the mental health of PWH.^[21]^ Our study did not find a significant difference in Black versus White race/ethnicity in pain severity, which supports the inconclusive evidence that MSM who identify as African American have elevated HIV incidence or related symptoms.^[21]^ The role of race and structural racism in regards to HIV should not be overlooked.^[21]^ Graduate school and higher compared with less than high school and being employed were also predictors of pain. This relationship is different from prior studies where lower education and lower employment rates were associated with pain.^[22]^ Employment status could be associated with pain if patients are working inside or outside the home that can cause mental or physical strain. Usually, PWH who have substantial pain report impairments in work,^[22]^ and future research should explain substance use and pain in PWH who are employed.

There were limitations to this cross-sectional study. First, our sample was predominantly insured MSM from 1 city who were enrolled in an alcohol treatment study. Despite potential reduced generalizability, a benefit of this approach is enhanced internal validity with validated measures of exposure and outcomes for all study participants. An additional limitation was the use of a single-item to measure pain severity and there are other pain scales that could be used to measure pain in greater detail.^[19]^ Patients with none or mild pain may report as such given, they are getting pain properly managed with the prescription opioids. In this cross-sectional study, we were not able to determine if the use of opioids resulted in reduction in pain, such that patients using opioids were more likely to report mild or no pain. Further research is needed to understand the use of opioids and pain management in PWH, and the implications of opioid use by patients with relatively low pain levels. Another limitation is that we did not obtain information about why our patients used substances. We report correlations, but due to the cross-sectional nature of the study we are unable to determine causes of substance use.

The relationship of depression to pain and use of these substances is important to understand among PWH. Prior research has found that PWH with moderate to severe pain are more likely to have depressive symptoms and use prescription opioids compared to those without pain.^[23]^ Furthermore, structural or social determinants of health such as homelessness and discrimination potentially precipitate psychiatric comorbidities and substance use for all PWH.^[21]^ Our population was mainly White PWH and future research should consider causes of pain in more diverse populations as pain is not well-studied among non-White PWH.^[24]^ Although our study sample was similar with respect to demographics of the overall clinic population, the sample was enrolled from a clinical trial. This limits generalizability, for example, to PWH who do not use alcohol. In the last decade, the prevalence of opioid use has changed, with a reduction in opioid prescribing and much higher use of illicit, new synthetic, and non-prescribed opioids. Furthermore, social determinants of health and incarceration have led to more drug injections resulting in negative consequences for PWH and the need for medications for opioid use disorder need to be scaled up.^[21]^

## 5. Conclusion

In summary, our results suggest that cannabis use and prescription opioid use are both commonly used in PWH experiencing moderate to severe pain, with very high pain prevalence among those using both substances (61%). A few socioeconomic factors, including education, and clinical factors (depression and anxiety) were also associated with moderate/high pain. With the aging population of PWH, pain is an increasingly common concern and routine pain assessment and screening for depression as well as potential problems associated with use of cannabis and prescription opioids should be considered.

## Acknowledgments

We are grateful to the study participants and to Kaiser Permanente for their support of HIV research.

## Author contributions

**Conceptualization:** Hannah J. Kim, Derek D. Satre, Michael J. Silverberg.

**Data curation:** Hannah J. Kim, Michael J. Silverberg.

**Formal analysis:** Hannah J. Kim.

**Funding acquisition:** Michael J. Silverberg, Derek D. Satre.

**Investigation:** Hannah J. Kim, Derek D. Satre, Michael J. Silverberg.

**Methodology:** Hannah J. Kim, Wendy Leyden, Amy S. Leibowitz.

**Resources:** Hannah J. Kim, Cynthia I. Campbell.

**Supervision:** Derek D. Satre, Michael J. Silverberg.

**Validation:** Derek D. Satre, Wendy Leyden, Amy S. Leibowitz, Cynthia I. Campbell

**Writing – original draft:** Hannah J. Kim.

**Writing – review & editing:** Hannah J. Kim, Derek D. Satre, Cynthia I. Campbell, Michael J. Silverberg.
